# Male and Female Mice Lacking Neuroligin-3 Modify the Behavior of Their Wild-Type Littermates

**DOI:** 10.1523/ENEURO.0145-17.2017

**Published:** 2017-07-31

**Authors:** Shireene Kalbassi, Sven O. Bachmann, Ellen Cross, Victoria H. Roberton, Stéphane J. Baudouin

**Affiliations:** School of Biosciences, Cardiff University, Cardiff CF10 3AX, Wales

**Keywords:** Autism spectrum disorders, neuroligin, parvalbumin interneurons, social behavior

## Abstract

In most mammals, including humans, the postnatal acquisition of normal social and nonsocial behavior critically depends on interactions with peers. Here we explore the possibility that mixed-group housing of mice carrying a deletion of *Nlgn3*, a gene associated with autism spectrum disorders, and their wild-type littermates induces changes in each other’s behavior. We have found that, when raised together, male *Nlgn3* knockout mice and their wild-type littermates displayed deficits in sociability. Moreover, social submission in adult male *Nlgn3* knockout mice correlated with an increase in their anxiety. Re-expression of *Nlgn3* in parvalbumin-expressing cells in transgenic animals rescued their social behavior and alleviated the phenotype of their wild-type littermates, further indicating that the social behavior of *Nlgn3* knockout mice has a direct and measurable impact on wild-type animals’ behavior. Finally, we showed that, unlike male mice, female mice lacking *Nlgn3* were insensitive to their peers’ behavior but modified the social behavior of their littermates. Altogether, our findings show that the environment is a critical factor in the development of behavioral phenotypes in transgenic and wild-type mice. In addition, these results reveal that the social environment has a sexually dimorphic effect on the behavior of mice lacking *Nlgn3*, being more influential in males than females.

## Significance Statement

Several studies have shown that the behavior and physiology of socially dominant mice differs from that of socially submissive mice. Despite this knowledge, no study has so far addressed the influence of group inequality, and in particular social hierarchy, on behavioral and Physiological measures obtained in mouse models of autism spectrum disorders and their wild-type littermates. In this study, we used a mouse model lacking the *Nlgn3* gene and demonstrated that, indeed, transgenic mice and their wild-type littermates can modify each other’s behavior. These observations could profoundly affect experiments using mouse models of psychiatric disorders, as they suggest that the use of wild-type littermate mice as controls may lead to misinterpretation of results.

## Introduction

In the majority of mammalian species, social groups are not egalitarian but organized in social hierarchies that can influence individuals’ behavior and stress. For example, in despotic hierarchies, the dominant individuals are more anxious than the submissive ones, whereas the contrary is found when the dominance is maintained through intimidation (Sapolsky, 2005). Inbred laboratory mice establish highly stable social hierarchies maintained through intimidation from the dominant male to the submissive males (Wang et al., 2011). Because of laboratory housing conditions, intimidations from dominant mice cannot be avoided, meaning that male dominance behavior is likely to raise anxiety levels in submissive individuals (Sapolsky, 2005). Indeed, studies show that social hierarchy can be a stressor for submissive animals and increase their motoric activity (Van Loo et al., 2003; Vargas-Pérez et al., 2009). Social rank also affects animals’ physiology; for example, socially submissive animals show decreased mRNA expression levels of corticotropin-releasing hormone receptor 2 and estrogen receptor α in the brain (Greenberg et al., 2014). Although mouse social hierarchies have only been well characterized in adult male laboratory mice (Wang et al., 2011), the potential existence of social hierarchies in groups of young or female laboratory mice cannot be excluded (Schuhr, 1987; Vom Saal et al., 1995; Garner et al., 2016).

Many mouse models for autism spectrum disorders (ASD) show social behavior defects and traits associated with social submission (Wang et al., 2011), namely courtship vocalization and territorial defects (Spencer et al., 2005; Kazdoba et al., 2016). The effect of social submission on phenotypes associated with ASD remains elusive but has been shown to influence courtship vocalization behavior of mice with a 16p11.2 microdeletion, a model for ASD (Yang et al., 2015a). Social environment can also have a positive impact on the phenotype of mouse models for ASD and rescue their sociability deficits (Yang et al., 2011). Although the effect of the social environment on phenotypes in mouse models for ASD is starting to be understood, the impact on the behavior of peers within the social housing group remains largely unknown. We speculated that rearing mouse models for ASD with nondeficient wild-type littermates might be sufficient to cause measurable behavioral and physiologic changes in all mice within the social environment.

Many genetic mouse models related to so-called syndromic forms of ASD display complex sets of phenotypes (Kazdoba et al., 2014; Portmann et al., 2014) not all directly related to autism. To minimize complexity, we have used a model of nonsyndromic ASD in which mice lack the X-linked gene *Nlgn3*, coding for the postsynaptic adhesion protein Neuroligin-3 exclusively expressed in the brain (Tanaka et al., 2010; Baudouin et al., 2012). In humans, *NLGN3* deletion is associated with nonsyndromic ASD (Jamain et al., 2003; Ylisaukko-oja et al., 2005; Levy et al., 2011; Sanders et al., 2011; C Yuen et al., 2017). The deletion of *Nlgn3* in mice leads to distinct measurable phenotypes, including social behavior and courtship deficits (Radyushkin et al., 2009; Fischer and Hammerschmidt, 2011; Baudouin et al., 2012; Rothwell et al., 2014). Moreover, behavioral phenotypes in *Nlgn3^y/–^* are mediated by deficits in the balance between excitation and inhibition in the striatum and long-term depression in the cerebellum (Baudouin et al., 2012; Rothwell et al., 2014; Zhang et al., 2015). Here, we show that male mice lacking *Nlgn3* are socially submissive to their wild-type littermates and that this social submission correlates with increased anxiety in *Nlgn3* knockout mice. We find that the behavior of male and female mice lacking *Nlgn3* modifies the social behavior of their littermates. Importantly, we show that re-expression of *Nlgn3* in parvalbumin-expressing interneurons in *Nlgn3^y/–^* mice rescues their social submission phenotype and the corresponding effect on the wild-type littermates, thus confirming that the behavior of mutant mice is causing the social behavior phenotype in their wild-type littermates.

## Materials and Methods

### Animals

**Figure 1.**
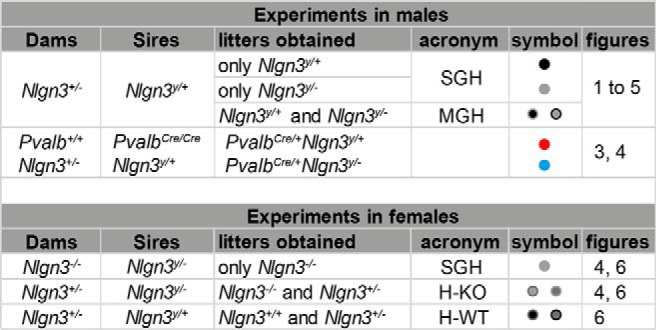
Summary of the breeding schemes.

**Figure 2.**
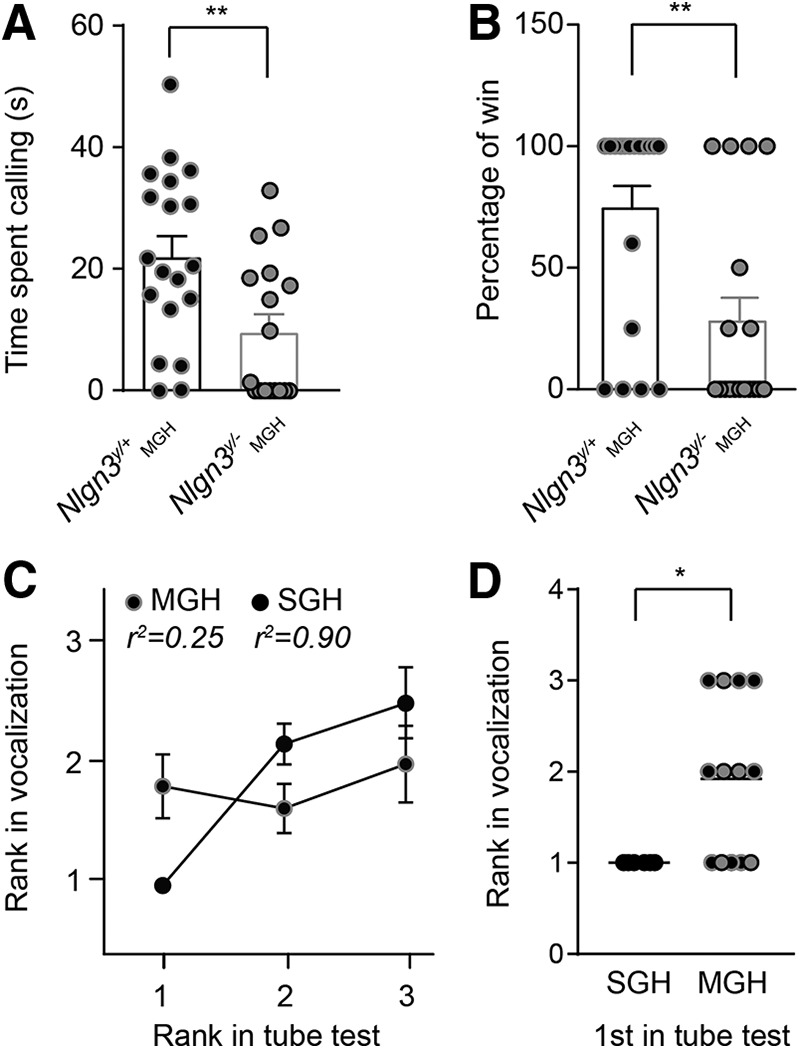
Adult *Nlgn3^y/–^* mice (2–4 mo old) are submissive to their *Nlgn3^y/+^* littermates. Littermate groups were composed of (1) *Nlgn3^y/+^* mice in SGH and (2) *Nlgn3^y/+^* and *Nlgn3^y/–^* mice in MGH. ***A***, *Nlgn3^y/–^* mice from MGH spent less time vocalizing to a female in estrus compared with *Nlgn3^y/+^* from MGH. ***B***, *Nlgn3^y/–^* mice from MGH lost more frequently in the tube test against their *Nlgn3^y/+^* littermates. ***C***, Absence of correlation between rank in the tube and ultrasonic vocalization tests in MGH mice. ***D***, For each group of mice from SGH and MGH, we plotted the rank in the vocalization test for the mice that were dominant in the tube test. All mice from SGH that won in the tube test also vocalized the most, and 5 of 13 mice from MGH (38%) that won in the tube test vocalized the most. For mice from MGH, black dots with gray circles are *Nlgn3^y/+^* mice, and gray dots with black circles are *Nlgn3^y/–^* mice. Values are represented as mean ± SEM. Statistical significance was tested by two-tailed *t* test (***A***) and two-tailed Mann–Whitney test (***B*** and ***C***); *P < 0.05; **P < 0.01.

**Figure 3.**
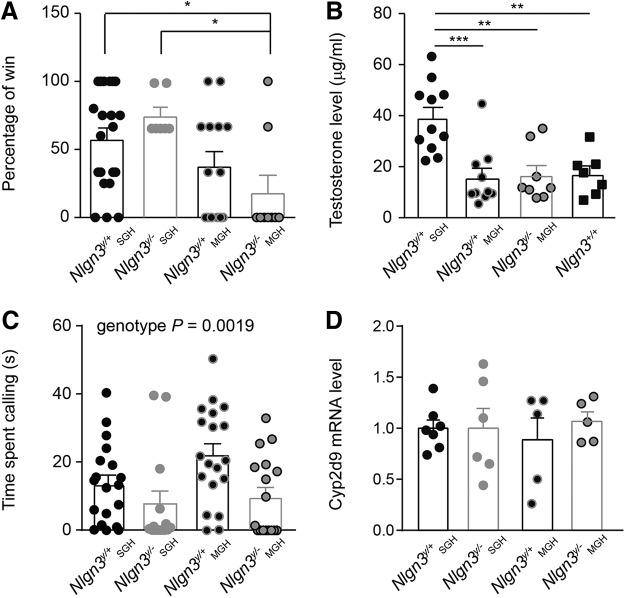
Decreased competitive behavior in adult (2- to 4-mo-old) *Nlgn3^y/–^* and *Nlgn3^y/+^* mice from MGH. ***A***, *Nlgn3^y/–^* mice from MGH were defeated more frequently than *Nlgn3^y/+^* and *Nlgn3^y/^* ^–^ mice from SGH when opposed to an unfamiliar submissive male in the tube test. ***B***, Increased urinary testosterone levels in *Nlgn3^y/+^* mice from SGH. ***C***, Housing conditions did not modify the time *Nlgn3^y/+^* or *Nlgn3^y/–^* mice spent calling a female in estrus. Regardless of the housing conditions, *Nlgn3^y/–^* mice spent less time calling a female in estrus than *Nlgn3^y/+^* mice. Note that data from Fig. 2*A* were replotted here to allow comparison. ***D***, Hepatic mRNA expression levels of *Cyp2d9* were similar between mice in MGH and SGH. Values are represented as mean ± SEM. Statistical significance was tested by Kruskal–Wallis test and *post hoc* Tukey’s multiple comparison test (***A*** and ***B***) and one-way ANOVA and *post hoc* Tukey’s multiple comparison test (***C*** and ***D***). *P < 0.05; **P < 0.01; ***P < 0.001.

**Figure 4.**
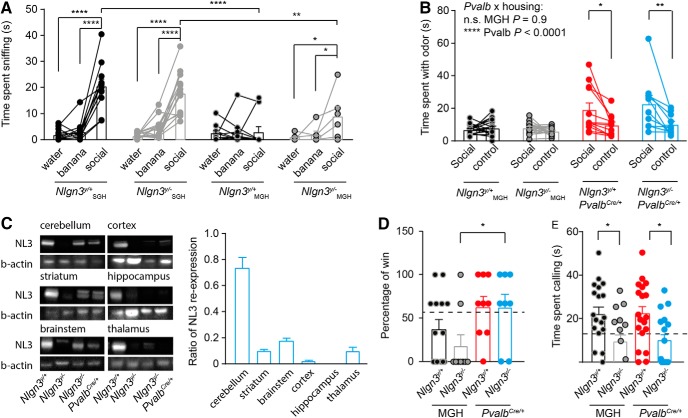
Adult (2- to 4-mo-old) *Nlgn3^y/–^* mice modify the social behavior of their littermates. ***A***, *Nlgn3^y/+^* and *Nlgn3^y/–^* mice from SGH and *Nlgn3^y/–^* mice from MGH showed increased interest in social odors compared with water, whereas this increased interest was absent in *Nlgn3^y/+^* mice from MGH. Note that *Nlgn3^y/+^* and *Nlgn3^y/–^* mice from SGH spent more time sniffing the social odor compared with *Nlgn3^y/+^* and *Nlgn3^y/–^* mice from MGH, respectively. ***B***, *Nlgn3^y/+^Pvalb^Cre/+^* and *Nlgn3^y/–^Pvalb^Cre/+^* mice showed an increased interest for social odors compared with *Nlgn3^y/+^* and *Nlgn3^y/–^* mice from MGH. ***C***, Western blot analysis shows a ratio of re-expression of 0.7 in the cerebellum, 0.15 in the brainstem, 0.1 in the striatum, 0.1 in the thalamus, and 0.04 in the cortex and no re-expression in the hippocampus. Note that the upper band in the brainstem *Nlgn3^y/–^* sample is most likely unspecific, as it appears in protein samples from *Nlgn3^y/–^* mice. ***D***, *Nlgn3^y/–^* and *Nlgn3^y/+^* mice from MGH lost more frequently against an unfamiliar submissive mouse compared with *Nlgn3^y/–^Pvalb^Cre/+^* and *Nlgn3^y/+^Pvalb^Cre/+^* mice. No difference in the percentage of wins against an unfamiliar submissive male was found in the tube test between *Nlgn3^y/+^Pvalb^Cre/+^* and *Nlgn3^y/–^Pvalb^Cre/+^* mice. Note that *Nlgn3^y/+^* and *Nlgn3^y/–^* mice from MGH data are replotted from Fig. 3*A*. The dotted line represents the mean percentage of wins for *Nlgn3^y/+^* mice from SGH. ***E***, *Nlgn3^y/–^* mice from MGH and *Nlgn3^y/–^Pvalb^Cre/+^* mice spent less time calling a female in estrus than *Nlgn3^y/+^* mice from MGH and *Nlgn3^y/+^Pvalb^Cre/+^*mice, respectively. The dotted line represents the mean time sent calling for *Nlgn3^y/+^* mice from SGH, replotted from Fig. 3*B*. Values are represented as mean ± SEM. Statistical significance was tested by two-way ANOVA repeated-measures (***A***), two-way ANOVA (***B***, ***D***, and ***E***), and *post hoc* Sidak’s multiple comparison test. *P < 0.05; **P < 0.001; ****P < 0.0001.

**Figure 5.**
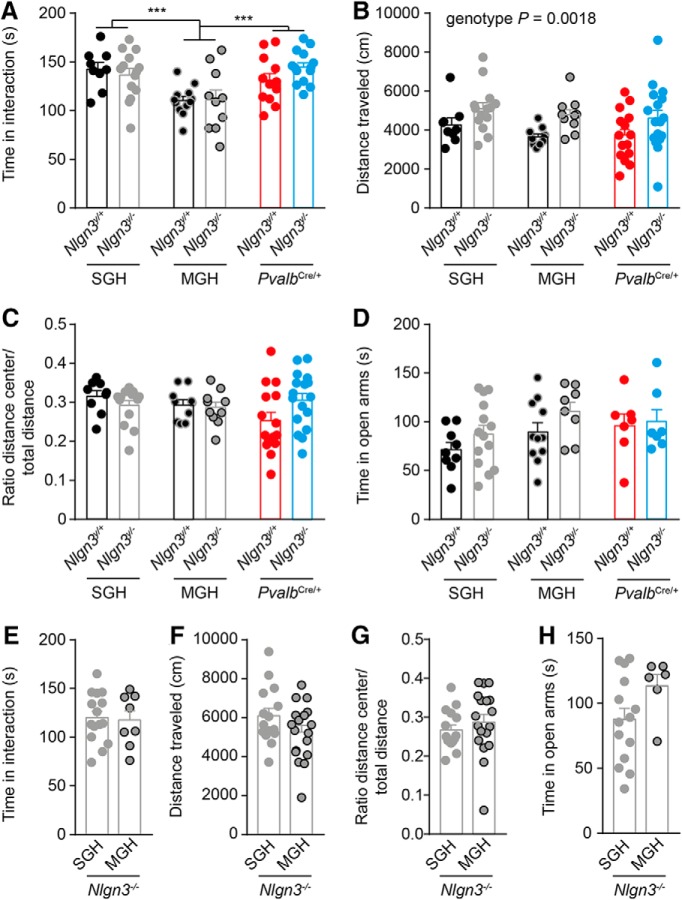
In young mice (P21–P28), MGH modifies the interest in social interaction of *Nlgn3^y/+^* and *Nlgn3^y/–^* but not *Nlgn3^–/–^*mice. ***A***, At P21–P28, *Nlgn3^y/+^* and *Nlgn3^y/–^* from SGH and *Nlgn3^y/+^Pvalb^Cre/+^* and *Nlgn3^y/–^Pvalb^Cre/+^* mice spent more time in contact with an unfamiliar female than *Nlgn3^y/+^* and *Nlgn3^y/–^* mice. ***B***, ***C***, No difference in the total distance and the normalized distanced traveled in the center of the OF. ***D***, No difference in the time spent in the open arms of the EPM. ***E–H***, MGH has no significant effect on the time spent in social interaction, the total distance and normalized distance traveled in the center of the OF, and the time spent in the open arm of the EPM of females lacking *Nlgn3* (*Nlgn3^–/–^*). Values are represented as mean ± SEM. Statistical significance was tested by two-way ANOVA and *post hoc* Sidak’s multiple comparison test (***A–C***). ***P < 0.001.

**Figure 6.**
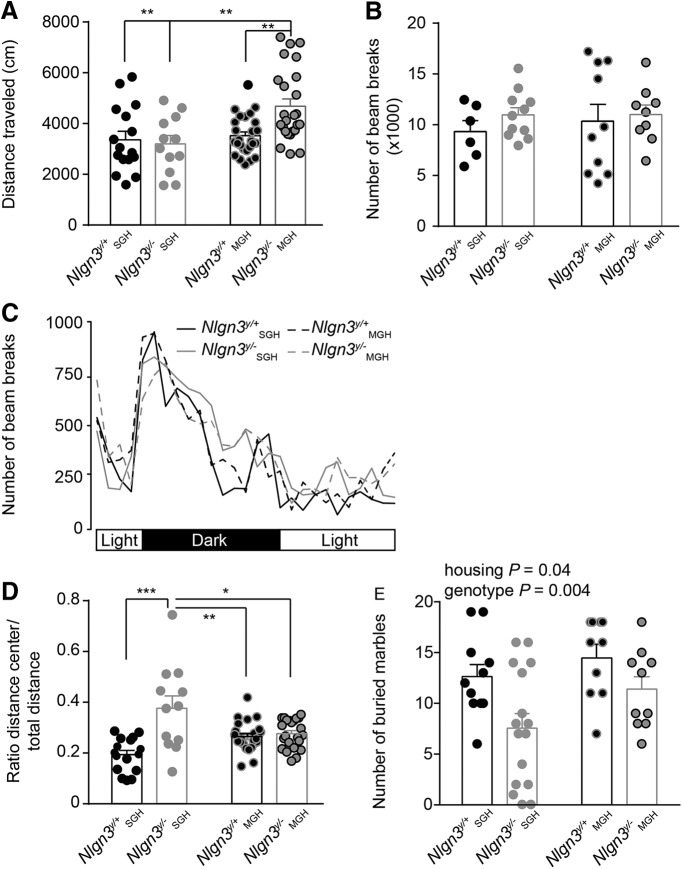
Increased anxiety in adult (2- to 4-mo-old) *Nlgn3^y/–^* mice from MGH. ***A***, Increased distance traveled in OF in *Nlgn3^y/–^* mice from MGH compared to *Nlgn3^y/–^* mice in SGH and *Nlgn3^y/+^*. ***B***, No change in the average number of beam breaks per hour recorded over 30 h. ***C***, Number of beam breaks per hour over 30 h. ***D***, Increased normalized distance traveled in the center of the OF for *Nlgn3^y/–^* mice in SGH compared with *Nlgn3^y/+^* mice from SGH and mice from MGH. ***E***, Increased number of marbles buried by mice in MGH compared with mice from SGH. *Nlgn3^y/–^* mice in SGH buried fewer marbles than *Nlgn3^y/+^* mice in SGH. Values are represented as mean ± SEM. Statistical significance was tested by two-way ANOVA and *post hoc* Sidak’s multiple comparison test. *P < 0.05; **P < 0.01; ***P < 0.001 (***A***, ***B***, ***D***, and ***E***).

**Figure 7.**
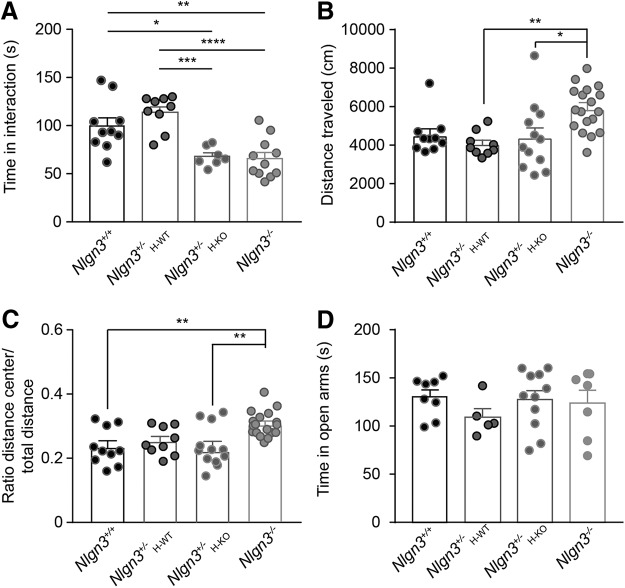
Adult (2- to 4-mo-old) *Nlgn3^–/–^* mice modify the social behavior of their littermates. We analyzed two littermate groups in which (1) *Nlgn3^+/–^* mice (*Nlgn3^+/–^*_H-WT_) were littermates of *Nlgn3^+/+^* mice or (2) *Nlgn3^+/–^* mice (*Nlgn3^+/–^*_H-KO_) were littermates of *Nlgn3^–/–^* mice. ***A***, *Nlgn3^+/–^*_H-KO_ and *Nlgn3^–/–^* mice spent less time in contact with an unfamiliar female than *Nlgn3^+/+^* and *Nlgn3^+/–^*_H-WT_ mice. ***B***, Increased distance traveled by *Nlgn3^–/–^* mice in the OF compared with *Nlgn3^+/–^*_H-WT_ and *Nlgn3^+/–^*_H-KO_ mice. ***C***, Increased normalized distance traveled in the center of the OF for *Nlgn3^–/–^* mice. ***D***, No change in the time spent in the open arms of the EPM. Values are represented as mean ± SEM. Statistical significance was tested by one-way ANOVA and *post hoc* Tukey’s multiple comparison test (***A***, ***C***, and ***D***) and Kruskal–Wallis and *post hoc* Dunn’s multiple comparison tests (***B***). *P < 0.05; **P < 0.001; ***P < 0.001; ***P < 0.0001.

All animal husbandry and experiments were performed in compliance with the UK Animals (Scientific Procedures) Act 1986, as amended, and in accordance with the Cardiff University animal care committee’s regulations. Mice containing a stop cassette flanked by loxP sites in the promoter region lacking *Nlgn3* expression (*Nlgn3^y/–^* #RBRC05451; Tanaka et al., 2010) and mice expressing Cre recombinase under the *Pvalb* endogenous promoter in *Pvalb*-expressing cells (*Pvalb^Cre/Cre^* mice JAX:017320; Hippenmeyer et al., 2005) were backcrossed to a C57Bl/6 background for at least eight generations. Male and female mice were separated at weaning but housed only with their own littermates. For a summary of the breeding schemes, refer to Fig. 1. Breeding between *Nlgn3^+/–^* mice and *Nlgn3^y^* ^/+^ mice produced 50% *Nlgn3^y/+^* and 50% *Nlgn3^y/^* ^–^ mice. We therefore obtained litters that contained both *Nlgn3^y/+^* and *Nlgn3^y/^* ^–^ mice [referred to as mixed genotype housing (MGH)] and, at a lower frequency, litters in which males were all of the same genotype, *Nlgn3^y/+^* or *Nlgn3^y/^* ^–^ mice [referred to as single genotype housing (SGH)]. Importantly, mice from SGH only ever encountered mice of the same genotype. *Nlgn3^+/–^* mice were crossed with *Nlgn3^y/+^ Pvalb^cre/cre^* mice to generate litters containing *Nlgn3^y/+^ Pvalb^cre/+^* and *Nlgn3^y/–^Pvalb^cre/+^* mice (Figs. 2–6). *Nlgn3^+/–^* mice were crossed with *Nlgn3^y/–^* males to generate litters containing *Nlgn3^–/–^* mice in MGH, and *Nlgn3^–/–^* mice were crossed with *Nlgn3^y/–^* males to generate litters containing *Nlgn3^–/–^* mice in SGH (Fig. 5). *Nlgn3^+/–^* mice were crossed with *Nlgn3^y^* ^/+^ male mice to generate litters containing *Nlgn3^+/^* ^+^ and *Nlgn3^+/–^*_H-WT_ mice, and *Nlgn3^+/–^* mice were crossed with *Nlgn3^y/–^* males to generate litters containing *Nlgn3^+/–^*_H-KO_ and *Nlgn3^–/–^* mice (Fig. 7). Sires were separated from pregnant dams, and mice were weaned at postnatal day 30 (P30) to avoid the potential confounds associated with weaning on mice tested at P21–P28.

Mice were kept on a 12-h light/dark cycle with free access to food and water. All behavior was assessed during the light cycle. Experiments in adult mice were conducted when mice were 2–4 mo old (Figs. 2–4, 6, and 7) and in young mice at P21–P28 (Fig. 5). To minimize anxiety associated with human handling, all mice were well handled before testing (Hurst and West, 2010). On the testing day, mice were habituated for 30 min to the testing room and handled with minimal restraint to reduce anxiety (Hurst and West, 2010). Tests were conducted over several days always in the following order: activity, marble burying, interest in social odors tests, courtship vocalization, and tube test. Note that all mice did not undergo testing in all tasks (refer to Statistical analysis).

### Interaction with females and ultrasonic vocalization

Before experiments, vaginal smears were stained with modified Giemsa solution (fixative and blue/azure dye) to determine the stage of estrus cycle (Caligioni, 2009). Test male mice were first habituated for 3 min to the arena. Subsequently, an unfamiliar female mouse in estrous was added to the same arena for 3 min. An experimenter blind to genotype manually scored interaction times. Interaction was recorded when mice were within 2 cm of each other. Ultrasonic vocalizations were recorded using an UltraSoundGate 416H preamplifier connected UltraSoundGate CM16 microphone (Aviosft Bioacoustics) and quantified automatically using SASLabPro software (Aviosft Bioacoustics). As previously described (Holy and Guo, 2005), events within the frequency range 30–200 Hz and longer than 5 ms were quantified, and the time spent calling was measured.

### Tube test

Social dominance within cages was assessed using the tube test apparatus (Noldus). This consists of a smooth transparent acrylic tube (length, 30 cm; internal diameter, 3.5 cm) with automated doors at both entrances and in the center. All mice were habituated to the tube for at least 10 min/d over at least 4 d. On testing days, mice were placed at opposite ends of the tube and released to enter the tube. When mice reached the center of the tube, the middle door opened and mice were challenged to push their opponent out of the tube. The mouse pushed out of the tube was declared the submissive mouse of that trial. To determine the cage hierarchy, mice from each cage were tested in pairs in a round-robin design, ensuring that all pairings of mice had been tested. The sides of entry for the mice were alternated to avoid bias. The test was repeated three times every 5 d, and the hierarchy on the third testing day was quantified. For testing using unfamiliar mice, we identified submissive C57Bl/6 mice using tube and ultrasonic vocalization tests before testing, and each test mouse encountered three different submissive mice. The percentage of wins, (number of wins)/(total number of encounter) × 100, was quantified.

### Spontaneous activity and elevated plus maze

Spontaneous activity of mice was recorded in a 40 × 20-cm open field (OF) arena for 20 min in the dark using an infrared video camera. EthoVision XT tracking software (Noldus) was used to measure the distance traveled in the OF (average centimeters traveled per second) and the normalized distance traveled in the center of the arena (5 cm from the wall), expressed as (distance traveled in the center of the OF)/(total distance traveled in the OF). For spontaneous activity over 30 h, mice were individually housed in clear cages (40 × 24 × 18 cm) with free access to food and water and maintained on their standard light/dark cycle. Three infrared beams passed the bottom part of the cage. Total beam breaks were recorded over 33 h, the first 3 h corresponding to a phase of habituation to the cages and the next 30 h to the testing phase. For the elevated plus maze (EPM), mice were placed for 5 min in a maze composed of four perpendicular 40-cm arms, two opposite arms with high walls, and two other opposite arms without walls. The exploration of the EPM was recorded by a computer-linked video camera located above the arena, and the time spent in the open arms was quantified using EthoVision XT.

### Interest in social odors

Social odors originated from two cages of three C57Bl/6 male mice with different parental origins, maintained for 6 d with the same home cage bedding to allow for a concentration of odorants. Before the test, swabs were wiped in a zigzag pattern across the bottom surface of the cage to collect the olfactory cues. Mice were acclimatized for 30 min to the presence of a cotton swab before testing. For the experiment presented in Fig. 4*A*, the following odors were presented in sequence: water, water, banana, banana, and social odor. During the 2-min exploration periods, the time spent sniffing the swab on the first exposure to each odor was recorded manually. For the experiment presented in Fig. 4*B*, a swab containing a social odor and a swab without odor were presented in two identical cups placed in opposite corners of the open field. Mice were able to be in direct contact with the odors, and exploration behavior was recorded over 4 min. The time spent in proximity to the social odor (<10 cm from the swab) was quantified using tracking software.

### Marble burying

Mice were placed in individual cages (28 × 17 cm) that each contained 20 marbles arranged in rows of four, on top of 4-cm-deep bedding, to allow burying behavior. The room used was dimly lit, with equal light distribution for all mice in the trial. In the days before testing, mice were habituated to the test cage for 30 min. On test days, mice were left for 30 min in the test cage containing the marbles. Marbles were manually counted and defined as buried when >50% of the marble was hidden. For quantification, experimenters were blind to the genotype.

### Testosterone dosage

Urine was collected from the mice immediately after bladder voiding and frozen instantly on dry ice. The urine was then stored at –20°C until testosterone quantification. Testosterone was quantified using ELISA in accordance with manufacturer instructions (Arbor Assays).

### RNA isolation and quantitative real-time PCR

Total RNA from liver was isolated with TRIzol reagent (Thermo Fisher Scientific) and purified using the RNeasy kit (Qiagen). cDNA was synthesized using Superscript III (Thermo Fisher Scientific). Quantitative real-time PCR analysis was performed using Fast SYBR green Master Mix (Thermo Fisher Scientific) on a real-time PCR System (Thermo Fisher Scientific). Relative expression levels were determined by normalization to 18S rRNA expression using the comparative ΔΔC_T_ method. Primers used were Cyp2d9 forward, 5′-AGTCTCTGGCTTAATTCCTGAT-3′, Cyp2d9 reverse, 5′-CGCAAGAGTATCGGGAATGC-3′, 18S forward, 5′-GTCTGTGATGCCCTTAGATG-3′, and 18S reverse′ 5′-AGCTTATGACCCGCACTTAC-3′.

### Western blot

Brain regions were dissected from adult mice after death and immediately frozen in liquid nitrogen. A volume of 100 ml lysis buffer was added to 10 mg tissue [50 mm Tris-HCl, 1 mm EDTA, 0.1% sodium dodecyl sulfate, 150 mm NaCl, 1% Triton X-100, 10 mm NaF, 1 mm NaVO_4_, 1 mm dithiothreitol, and complete protease inhibitor (Sigma-Aldrich)]. The amount of protein in each sample was quantified using a bicinchoninic acid protein assay. Samples were then diluted in lithium dodecyl sulfate buffer (106 mm Tris-HCl, 141 mm Tris-base, 2% lithium dodecyl sulfate, 10% glycerol, 0.51 mm EDTA, 0.22 mm G250 Coomassie Blue, 0.175 mm Phenol Red, and 10 mm DTT, pH 8.5), and 25 mg of each sample was loaded onto 4–12% Bis-Tris polyacrylamide gels and transferred onto nitrocellulose membranes. Rabbit monoclonal anti–Neuroligin-3 (Abcam; clone EPR16158) and chicken anti–β-actin (Abcam; clone 2G10) antibodies were used. Signals were detected using horseradish peroxidase and Alexa Fluor 488–conjugated secondary antibodies (Thermo Fisher Scientific). The level of re-expression was quantified as (Neuroligin-3/actin) in *Nlgn3^y/–^Pvalb^Cre/+^* mice normalized to (Neuroligin-3/actin) in *Nlgn3^y/+^* mice.

### Statistical analysis

Table 1 contains the data structure, type of test used, observed power, and *n* for each figure. Each test included enough animals to reach a power close to or higher than 0.8. Mice were not systematically tested for all tasks. We designed our test groups to have mice from the different groups tested at the same time. We used GraphPad Prism to systematically test for normality using the D’Agostino–Pearson test and for outliers using the regression and outlier removal (ROUT) method with *Q* = 1 to ensure that no outliers would modify the outcome and power of the statistical tests. No animals were removed from the analyses. For each experiment, at least three independent litters were analyzed. Pairwise comparisons were analyzed by two-tailed Student’s *t* test for normally distributed datasets or two-tailed Mann–Whitney test for non–normally distributed datasets. Multiple comparisons were performed using one-way ANOVA for normally distributed datasets or Mann–Whitney test for non–normally distributed datasets, followed by Tukey’s or Dunn’s *post hoc* tests for multiple comparisons when appropriate. All datasets used for two-way nonrepeated and repeated-measure ANOVAs were normally distributed and, when appropriate, followed by *post hoc* Sidak’s test. All statistical data are presented as mean ± SEM.

**Table 1.** Details of statistical analysis

Figure	Data structure	Type of test	Observed power	*n*
2*A*	Normal distribution	*t* test	0.78	*Nlgn3^y/+^* MGH: 19*Nlgn3^y/–^* MGH: 17
2*B*	Nonparametric	Mann–Whitney	NA	*Nlgn3^y/+^* MGH: 19*Nlgn3^y/–^* MGH: 17
2*C*	Unknown	Correlation test	NA	MGH: 13 cagesSGH: 6 cages
2*D*	Non parametric	Mann–Whitney	NA	MGH: 13 cagesSGH: 6 cages
3*A*	Nonparametric	Kruskal–Wallis	NA	*Nlgn3^y/+^* SGH: 21*Nlgn3^y/–^* SGH: 7*Nlgn3^y/+^* MGH: 14*Nlgn3^y/–^* MGH:9
3*B*	Normal distribution	One way ANOVA/Tukey’s multiple comparison	0.99	*Nlgn3^y/+^* SGH: 11*Nlgn3^y/+^* MGH: 10*Nlgn3^y/–^* MGH: 8*Nlgn3^+/+^*: 7
3*C*	Normal distribution	Two-way ANOVA	Genotype 0.896	*Nlgn3^y/+^* SGH: 19*Nlgn3^y/+^* SGH: 14*Nlgn3^y/+^* MGH: 19*Nlgn3^y/–^* MGH: 17
3*D*	Normal distribution	One-way ANOVA	NA	*Nlgn3^y/+^* SGH: 7*Nlgn3^y/–^* SGH: 6*Nlgn3^y/+^* MGH: 5*Nlgn3^y/–^* MGH: 5
4*A*	Normal distribution	Two-way ANOVA repeated measure/Sidak’s multiple comparison/within-subject effect	Interaction odor × genotype 1	*Nlgn3^y/+^* SGH: 12*Nlgn3^y/–^* SGH: 17*Nlgn3^y/+^* MGH: 9*Nlgn3^y/–^* MGH: 6
			Genotype 1	
4*B*	Normal distribution	Two-way ANOVA/Sidak’s multiple comparison	Pvalb 1	*Nlgn3^y/+^* MGH: 18*Nlgn3^y/–^* MGH: 19*Nlgn3^y/+^ Pvalb* ^Cre/+^: 7*Nlgn3^y/–^ Pvalb* ^Cre/+^:7
			Odor 0.99	
			Interaction PV × odor 0.94	
4*C*	Normal distribution	NA	NA	*Nlgn3^y/+^*: 6*Nlgn3^y/–^ Pvalb* ^Cre/+^:7
4*D*	Normal distribution	Two-way ANOVA	Pvalb 0.77	*Nlgn3^y/+^* MGH: 14*Nlgn3^y/–^* MGH:9*Nlgn3^y/+^ Pvalb* ^Cre/+^: 9*Nlgn3^y/–^ Pvalb* ^Cre/+^: 8
4*E*	Normal distribution	Two-way ANOVA	Genotype 0.78	*Nlgn3^y/+^* MGH: 19*Nlgn3^y/–^* MGH: 17*Nlgn3^y/+^ Pvalb* ^Cre/+^: 11*Nlgn3^y/–^ Pvalb* ^Cre/+^: 10
5*A*	Normal distribution	Two-way ANOVA/Sidak’s multiple comparison	Housing 0.984	*Nlgn3^y/+^* SGH: 9*Nlgn3^y/–^* SGH: 14*Nlgn3^y/+^* MGH: 10*Nlgn3^y/–^* MGH: 10*Nlgn3^y/+^ Pvalb* ^Cre/+^:15*Nlgn3^y/–^ Pvalb* ^Cre/+^: 17
5*B*	Normal distribution	Two-way ANOVA	NA	*Nlgn3^y/+^* SGH: 8*Nlgn3^y/–^* SGH: 9*Nlgn3^y/+^* MGH: 12*Nlgn3^y/–^* MGH: 10*Nlgn3^y/+^ Pvalb* ^Cre/+^:15*Nlgn3^y/–^ Pvalb* ^Cre/+^: 17
5*C*	Nonparametric	Kruskal–Wallis	NA	*Nlgn3^y/+^* SGH: 9*Nlgn3^y/–^* SGH: 14*Nlgn3^y/+^* MGH: 10*Nlgn3^y/–^* MGH: 10*Nlgn3^y/+^ Pvalb* ^Cre/+^:15*Nlgn3^y/–^ Pvalb* ^Cre/+^: 17
5*D*	Normal distribution	Two-way ANOVA	NA	*Nlgn3^y/+^* SGH: 9*Nlgn3^y/–^* SGH: 14*Nlgn3^y/+^* MGH: 11*Nlgn3^y/–^* MGH: 8*Nlgn3^y/+^ Pvalb* ^Cre/+^:12*Nlgn3^y/–^ Pvalb* ^Cre/+^: 7
5*E*	Normal distribution	*t* test	NA	*Nlgn3^–/–^* SGH: 15*Nlgn3^–/–^* MGH: 8
5*F*	Normal distribution	*t* test	NA	*Nlgn3^–/–^* SGH: 15*Nlgn3^–/–^* MGH: 18
5*G*	Nonparametric	Mann–Whitney	NA	*Nlgn3^–/–^* SGH: 15*Nlgn3^–/–^* MGH: 18
5*H*	Normal distribution	*t* test	NA	*Nlgn3^–/–^* SGH: 14*Nlgn3^–/–^* MGH: 6
6*A*	Normal distribution	Two-way ANOVA/Sidak’s multiple comparison	Housing 0.83Interaction 0.672	*Nlgn3^y/+^* SGH: 16*Nlgn3^y/–^* SGH: 12*Nlgn3^y/+^* MGH: 26*Nlgn3^y/–^* MGH: 25
6*B*-*C*	Normal distribution	Two-way ANOVA	NA	*Nlgn3^y/+^* SGH: 6*Nlgn3^y/–^* SGH: 11*Nlgn3^y/+^* MGH: 10*Nlgn3^y/–^* MGH: 9
6*B*	Normal distribution	Two-way ANOVA/Sidak’s multiple comparison	Genotype 0.997Interaction 0.987	*Nlgn3^y/+^* SGH: 16*Nlgn3^y/–^* SGH: 12*Nlgn3^y/+^* MGH: 26*Nlgn3^y/–^* MGH: 25
6*E*	Normal distribution	Two-way ANOVA/Sidak’s multiple comparison	Genotype 0.76Genotype 0.52	*Nlgn3^y/+^* SGH: 11*Nlgn3^y/–^* SGH: 16*Nlgn3^y/+^* MGH: 10*Nlgn3^y/–^* MGH: 10
7*A*	Normal distribution	One way ANOVA/Tukey’s multiple comparison	0.99	*Nlgn3^+/+^*: 10*Nlgn3^+/–^*_H-WT_: 9*Nlgn3^+/–^*_H-KO_: 7*Nlgn3^–/–^*: 11
7*B*	Nonparametric	Kruskal–Wallis/Dunn’s multiple comparison	0.94	*Nlgn3^+/+^*: 10*Nlgn3^+/–^*_H-WT_: 9*Nlgn3^+/–^*_H-KO_: 12*Nlgn3^–/–^*: 18
7*C*	Normal distribution	One way ANOVA/Tukey’s multiple comparison	0.96	*Nlgn3^+/+^*: 10*Nlgn3^+/–^*_H-WT_: 9*Nlgn3^+/–^*_H-KO_: 12*Nlgn3^–/–^*: 18
7*D*	Normal distribution	One way ANOVA	NA	*Nlgn3^+/+^*: 8*Nlgn3^+/–^*_H-WT_: 5*Nlgn3^+/–^*_H-KO_: 11*Nlgn3^–/–^*: 7

NA, not applicable.

## Results

To investigate the role of social hierarchy in the behavior of *Nlgn3^y/–^* mice and their wild-type (*Nlgn3^y/+^*) littermates, we analyzed the behavior of mice from litters consisting of both genotypes (MGH) in comparison to litters in which male mice were all of the same *Nlgn3^y/+^* genotype (SGH). As previously reported (Radyushkin et al., 2009; Fischer and Hammerschmidt, 2011), *Nlgn3^y/–^* mice from MGH emitted fewer ultrasonic vocalizations during courtship in comparison to *Nlgn3^y/+^* from MGH [*Nlgn3^y/+^* 22.2 ± 3.5 s and *Nlgn3^y/–^* 9.8 ± 2.8 s, *P* = 0.007, *F*_(18,16)_ = 1.49; Fig. 2*A*]. Additionally, in the tube test, *Nlgn3^y/–^* mice from MGH lost more frequently when competing against *Nlgn3^y/+^* mice from MGH (*Nlgn3^y/+^* 72.9% ± 9.8% and *Nlgn3^y/–^* 29.4% ± 10.3%; *P* = 0.0074; Fig. 2*B*), suggesting that they are socially submissive to their wild-type littermates (Wang et al., 2011). In groups of mice with a stable social hierarchy, there is a correlation between ranking in courtship vocalization and in the tube test (Wang et al., 2011). The dominant mouse vocalizes the most and wins in the tube test, and the most submissive mouse vocalizes the least and is defeated more often in the tube test. An examination of social hierarchy in SGH mice compared with MGH mice revealed a linear correlation between tube test and courtship rankings for mice in SGH but not for mice in MGH (Fig. 2*C*). In particular, all mice from SGH that won in the tube test were the ones vocalizing the most (SGH 1 and MGH 1.92, *P* = 0.033; Fig. 2*D*), indicating a clear social hierarchy. However, only 38% of the mice from MGH that won the tube test were the ones that vocalized the most (Fig. 2*D*), revealing that a structured social hierarchy had not developed in mice from MGH.

Because of this defect in the development of a social hierarchy in mice from MGH, we compared their territorial and courtship behavior with that of *Nlgn3^y/+^* mice from SGH. In the tube test, unfamiliar submissive male mice more frequently defeated *Nlgn3^y/–^* mice from MGH than *Nlgn3^y/+^* and *Nlgn3^y/–^* mice from SGH (*Nlgn3^y/+^* SGH 57.9% ± 7.9%, *Nlgn3^y/–^* SGH 76.2% ± 6.1%, *Nlgn3^y/+^* MGH 38.1% ± 10.4%, and *Nlgn3^y/–^* MGH 18.5% ± 12.6%, *P* = 0.023; Fig. 3*A*). Levels of testosterone, a marker of social dominance (Juntti et al., 2010), were also reduced in the urine of *Nlgn3^y/–^* and *Nlgn3^y/+^* mice from MGH compared with those of *Nlgn3^y/+^* mice from SGH and unexpectedly not different from levels found in the urine of *Nlgn3^+/+^* mice [*Nlgn3^y/+^* SGH 39,223 ± 4106 µg/ml, *Nlgn3^y/+^* MGH 15,738 ± 3687 µg/ml, *Nlgn3^y/–^* MGH 16,736 ± 3779 µg/ml, and *Nlgn3^+/+^* 17,182 ± 3160 µg/ml, main effect *P* < 0,0001, *F*_(3,32)_ = 9.85; Fig. 3*B*]. The time spent calling a female in estrus was similar between *Nlgn3^y/+^* mice from SGH and MGH and increased compared with *Nlgn3^y/–^* mice from SGH and MGH [*Nlgn3^y/+^* SGH 13.6 ± 2.7 s, *Nlgn3^y/–^* SGH 6.38 ± 3.2 s, *Nlgn3^y/+^* MGH 22.2 ± 3.5 s, and *Nlgn3^y/–^* MGH 9.8 ± 2.8 s; main effect, *P* = 0.014, *F*_(2,52)_ = 4.62; Fig. 3*C*]. Note that the expression level of *Cyp2d9*, a marker of sexual dimorphism in the liver, was not affected by housing (*Nlgn3^y/+^* SGH 1 ± 0.08, *Nlgn3^y/–^* SGH 0.88 ± 0.21, *Nlgn3^y/+^* MGH 0.89 ± 0.21, and *Nlgn3^y/–^* MGH 1.07 ± 0.09; Fig. 3*D*), indicating a similar degree of sexual maturation between the different groups of mice. These results demonstrate that social housing modifies the competitive behavior of *Nlgn3^y/–^* mice without affecting their courtship behavior.

Because social hierarchy is affected in litters from MGH, we speculated that the social behavior of *Nlgn3^y/+^* and *Nlgn3^y/–^* mice from MGH would also be affected, and therefore investigated their interest for social odors. Whereas *Nlgn3^y/+^* mice from SGH spent more time investigating social cues compared with water or banana odors, *Nlgn3^y/+^* mice from MGH spent a similar amount of time investigating social and nonsocial cues, showing an absence of interest for social cues [*Nlgn3^y/+^* SGH: water 1.9 ± 0.6 s, banana 3.1 ± 1.1 s, social 20.6 ± 2.6 s; *Nlgn3^y/–^* SGH: water 1.4 ± 0.2 s, banana 4.0 ± 0.9 s, social 17.8 ± 1.9 s; *Nlgn3^y/+^* MGH: water 2.6 ± 1 s, banana 2.8 ± 5.2 s, social 3 ± 2 s; *Nlgn3^y/–^* MGH: water 0.7 ± 0.5 s, banana 2 ± 1.3 s, social 8.96 ± 1.9 s; main effect of odor, *P* = 0.0001, *F*_(2,82)_ = 57.7; main effect of genotype, *P* = 0.0006, *F*_(3,41)_ = 7.0; interaction odor × genotype, *P* < 0.0001, *F*_(6,82)_ = 9.7; Fig. 4*A*]. In addition, *Nlgn3^y/+^* mice from MGH spent a similar amount of time exploring social odors and control presented simultaneously, showing that their social discrimination was also affected [*Nlgn3^y/+^* MGH: social 7 ± 0.7 s, control 8 ± 1.2 s; *Nlgn3^y/–^* MGH: social 8 ± 0.8 s, control 6 ± 0.6 s; *Nlgn3^y/+^Pvalb^Cre/+^*: social 18 ± 6.4 s, control 6.3 ± 1.7 s; *Nlgn3^y/–^Pvalb^Cre/+^*: social 17.4 ± 3.6 s, control 9.1 ± 2.5 s; main effect of odor, *P* < 0.0001, *F*_(1110)_ =15.6; main effect of genotype, *P* < 0.0001, *F*_(3110)_ = 10.7; interaction *Pvalb* × odor, *P* < 0.0001, *F*_(1.114)_ = 31.9; Fig. 4*B*]. Although *Nlgn3^y/–^* mice from SGH and *Nlgn3^y/–^* from MGH spent more time investigating social cues, *Nlgn3^y/–^* mice from SGH spent more time investigating the social odor than *Nlgn3^y/–^* mice from MGH. Note that *Nlgn3^y/–^* mice from MGH showed more interest in social odor than banana (Fig. 4*A*) but failed to show a preference for social odors versus control (Fig. 4*B*). This is most likely due to differences in protocol: Fig. 4*A* measures the interest for odors and Fig. 4*B* the ability to discriminate between social and nonsocial cues (see Materials and Methods). These results indicate that MGH affects the interest in social odors of *Nlgn3^y/+^* and *Nlgn3^y/–^* mice.

To investigate the causal role of *Nlgn3^y/–^* mouse behavior in that of their littermates, we re-expressed *Nlgn3* in parvalbumin (*Pvalb*)-expressing interneurons, known to regulate social behavior in mice (Belforte et al., 2010; Del Pino et al., 2013; Saunders et al., 2013; Billingslea et al., 2014; Ito-Ishida et al., 2015; Wöhr et al., 2015; Zou et al., 2016). After *Nlgn3* re-expression in *Pvalb*-expressing interneurons, we detected Neuroligin-3 in several parts of the brain, including cerebellum (ratio *Nlgn3^y/–^Pvalb^Cre/+^* to *Nlgn3^y/+^*: ∼0.7), brainstem (∼0.15), striatum (∼0.1), thalamus (∼0.1), and cortex (∼0.04) and failed to detect it in the hippocampus (Fig. 4*C*). Re-expression of *Nlgn3* in *Pvalb*-expressing interneurons was sufficient to increase the time *Nlgn3^y/–^Pvalb^Cre/+^* and *Nlgn3^y/+^Pvalb^Cre/+^* mice spent investigating social odors compared with controls (Fig. 4*B*). In addition, we found that *Nlgn3^y/–^Pvalb^Cre/+^* mice won as frequently against submissive wild-type mice but vocalized less than *Nlgn3^y/+^Pvalb^Cre/+^* mice (*Nlgn3^y/+^Pvalb^Cre/+^* 62.9% ± 11.7%, *Nlgn3^y/–^Pvalb^Cre/+^* 62.5% ± 14.7%; Fig. 4*D*; *Nlgn3^y/+^Pvalb^Cre/+^* 23.5 ± 3.3 s, *Nlgn3^y/–^Pvalb^Cre/+^* 8.4 ± 5.2 s; *P* = 0.01; Fig. 4*E*), indicating that *Nlgn3* in *Pvalb*-expressing interneurons controls the territorial behavior but not courtship behavior in individual mice. To determine whether re-expression of *Nlgn3* in *Pvalb*-expressing cells affects social interaction in *Nlgn3^y/–^* and *Nlgn3^y/+^* mice, we exposed mice at P21–P28 to an unfamiliar adult female mouse and measured the amount of time they spent interacting. *Nlgn3^y/–^* mice and their *Nlgn3^y/+^* littermates spent less time in social interaction than *Nlgn3^y/+^* and *Nlgn3^y/–^* mice from SGH or *Nlgn3^y/–^Pvalb^Cre/+^* and *Nlgn3^y/+^Pvalb^Cre/+^* mice [*Nlgn3^y/+^* SGH 142.6 ± 6.8 s, *Nlgn3^y/–^* SGH 136.6 ± 6.6 s, *Nlgn3^y/+^* MGH 110.3 ± 4.4 s, *Nlgn3^y/–^* MGH 111 ± 10.2 s, *Nlgn3^y/–^Pvalb^Cre/+^* 131.1 ± 7 s, *Nlgn3^y/+^Pvalb^Cre/+^* 142 ± 5.6 s; main effect of housing *P* < 0.0001, *F*_(2,65)_ = 11.4; *P* < 0.001 in Fig. 5*A*], indicating that re-expression of *Nlgn3* in *Pvalb*-expressing cells rescues the interest in social interaction of *Nlgn3^y/–^* and *Nlgn3^y/+^* mice to levels similar to those of mice from SGH. Independently of the housing and re-expression in *Pvalb-*expressing interneurons, *Nlgn3^y/–^* mice traveled greater total distance in the OF than *Nlgn3^y/+^* mice [*Nlgn3^y/+^* SGH 3977 ± 189 cm, *Nlgn3^y/–^* SGH 4753 ± 202 cm, *Nlgn3^y/+^* MGH 3796 ± 186 cm, *Nlgn3^y/–^* MGH 5299 ± 426 cm, *Nlgn3^y/–^Pvalb^Cre/+^* 3683 ± 314 cm, *Nlgn3^y/+^Pvalb^Cre/+^* 4856 ± 363 cm; main effect of genotype *P* = 0.0018, *F*_(1,69)_ = 10.49; Fig. 5*B*]. Mice from all genotype and housing groups traveled a similar normalized distance in the center of the OF (*Nlgn3^y/+^* SGH 0.32 ± 0.02 cm, *Nlgn3^y/–^* SGH 0.29 ± 0.01 cm, *Nlgn3^y/+^* MGH 0.29 ± 0.01 cm, *Nlgn3^y/–^* MGH 0.29 ± 0.01 cm, *Nlgn3^y/–^Pvalb^Cre/+^* 0.25 ± 0.02 cm, *Nlgn3^y/+^Pvalb^Cre/+^* 0.31 ± 0.02 cm; Fig. 5*C*). Mice from all genotype and housing groups spent similar amounts of time in the open arms of the EPM (*Nlgn3^y/+^* SGH 71.3 ± 7.5 s, *Nlgn3^y/–^* SGH 87.26 ± 9.0 s, *Nlgn3^y/+^* MGH 89.6 ± 9.6 s, *Nlgn3^y/–^* MGH 110.5 ± 9.6 s, *Nlgn3^y/–^Pvalb^Cre/+^* 95.9 ± 12.1 s, *Nlgn3^y/+^Pvalb^Cre/+^* 100.4 ± 12.2 s; Fig. 5*D*). These results show that *Nlgn3^y/–^* and *Nlgn3^y/+^* mice from MGH are not more anxious than mice from SGH. Note that there are no significant differences between the behavior of *Nlgn3^y/+^* mice from SGH or MGH and *Nlgn3^y^*^/^*^+^Pvalb* ^Cre/+^ mice (Fig. 4*D*, *E* and Fig. 5*A–D*). This indicates that, consistent with previous reports, the expression of Cre recombinase in parvalbumin-expressing neurons of wild-type animals is not sufficient to produce a phenotype in wild-type male mice (Hippenmeyer et al., 2005). In contrast to *Nlgn3^y/–^* mice, female mice from MGH lacking *Nlgn3* (*Nlgn3^–/–^*) spent a similar amount of time in social contact with *Nlgn3^–/–^* mice from SGH (SGH 120 ± 6.6 s and MGH 117.5 ± 9.4 s; Fig. 5*E*). *Nlgn3^–/–^* mice from MGH and from SGH traveled a similar distance in the OF (SGH 6098 ± 379 cm and MGH 5253 ± 338 cm; Fig. 5*F*), similar normalized distance in the center of the OF (SGH 0.27 ± 0.01 cm and MGH 0.29 ± 0.02 cm; Fig. 5*G*), and similar amounts of time in the open arms of the EPM (SGH 87.3 ± 9.0 s and MGH 113 ± 9.0 s; Fig. 5*H*). These results demonstrate that MGH modifies the social behavior of *Nlgn3^y/+^* and *Nlgn3^y/–^* mice but not that of *Nlgn3^–/–^* mice. Moreover, re-expression of *Nlgn3* in *Pvalb*-expressing interneurons restores social behavior in *Nlgn3^y/–^* mice and their wild-type littermates to levels found in mice from SGH, demonstrating that *Nlgn3^y/–^* mice modify the social behavior of their littermates.

The social submission of *Nlgn3^y/–^* mice could modify their anxiety, so we next examined the effect of social housing conditions on stress related to a novel environment and to burying behavior. To investigate this anxiety, we analyzed the behavior of mice from litters consisting of both genotypes (MGH), in comparison with litters in which male mice were all of the same genotype, *Nlgn3^y/+^* or *Nlgn3^y/–^* (SGH). Radyushkin et al. (2009) showed that *Nlgn3^y/–^* mice are hyperactive and less stressed than their *Nlgn3^y/+^* littermates. Importantly, *Nlgn3^y/–^* mice showed increased activity in the OF, increased exploration of the hole board, and decreased burying behavior but not change in the exploration of the EPM, indicating that only certain aspects of their anxiety were affected (Radyushkin et al., 2009). Consistently, we found that *Nlgn3^y/–^* mice from MGH traveled further in the OF compared with *Nlgn3^y/+^* mice from MGH [*Nlgn3^y/+^* SGH 3373 ± 323 cm, *Nlgn3^y/–^* SGH 3186 ± 324 cm, *Nlgn3^y/+^* MGH 3521 ± 151 cm, and *Nlgn3^y/–^* MGH 4684 ± 285 cm; main effect of housing *P* = 0.004, *F*_(1,75)_ = 8.82; interaction housing × genotype *P* = 0.017, *F*_(1,75)_ = 5.94; Fig. 6*A*]. Although housing condition had no effect on *Nlgn3^y/+^* mice, *Nlgn3^y/–^* mice from MGH traveled an increased distance in comparison to *Nlgn3^y/–^* from SGH (Fig. 6*A*). However, spontaneous activity (number of beam break) over 30 h (*Nlgn3^y/+^* SGH 9322 ± 1090, *Nlgn3^y/–^* SGH 10,979 ± 703, *Nlgn3^y/+^* MGH 10,357 ± 1648 and *Nlgn3^y/–^* MGH 11,005 ± 933; Fig. 6*B*) and increased nocturnal activity (Fig. 6*C*) did not differ from any other group tested. Increased velocity in an OF can be triggered by an increased motoric drive and by emotional reactivity to the novelty of the environment. The time spent by the wall of the open field (thigmotaxis) is a measure of this anxiety. To correct for the differing distances traveled between groups, we normalized the distance traveled in the center to the total distance traveled by the mice in the OF. *Nlgn3^y/–^* mice from SGH showed decreased thigmotaxis compared with *Nlgn3^y/–^* from MGH, whereas housing condition had no effect on the thigmotaxis (ratio distance in the center/total distance) of *Nlgn3^y/+^*mice [*Nlgn3^y/+^* SGH 0.19 ± 0.01, *Nlgn3^y/–^* SGH 0.37 ± 0.04, *Nlgn3^y/+^* MGH 0.26 ± 0.01, and *Nlgn3^y/–^* MGH 0.28 ± 0.02; main effect of genotype *P* < 0.0001, *F*_(1,72)_ = 22.35; interaction genotype × housing *P* < 0.0001, *F*_(1,72)_= 17.29; Fig. 6*D*]. In addition, we found that mice from MGH buried more marbles than mice from SGH [*Nlgn3^y/+^* SGH 12.6 ± 1.2, *Nlgn3^y/–^* SGH 7.6 ± 1.4, *Nlgn3^y/+^* MGH 14.6 ± 3.9, and *Nlgn3^y/–^* MGH 11.4 ± 1.2; main effect of housing *P* = 0.0407, *F*_(1,43)_ = 4.45; main effect of genotype *P* = 0.0044, *F*_(1,43)_ = 9.06; Fig. 6*E*]. Increased numbers of buried marbles can be a result of increased anxiety or increased repetitive behavior (Deacon, 2006). Therefore, these results show that mixed genotype housing increases anxiety or compulsive behavior in adult *Nlgn3^y/–^* and, to a lesser extent, *Nlgn3^y/+^* mice. These results also show that *Nlgn3^y/–^* mice are more reactive to novelty when raised in MGH compared with SGH, indicating that anxiety related to novelty is increased in these mice when raised with wild-type littermates.

We then investigated whether females lacking *Nlgn3* would show behavior similar to that of *Nlgn3^y/–^* mice and, in particular, influence the behavior of their littermates. Because *Nlgn3* is an X-linked gene, we could not naturally obtain littermate cages containing *Nlgn3^–/–^* and *Nlgn3^+/+^* mice. Therefore, we investigated the effect of *Nlgn3^–/–^* or *Nlgn3^+/+^* mouse behavior on that of *Nlgn3^+/–^* littermates. We found that *Nlgn3^+/–^* mice raised with *Nlgn3^+/+^* littermates were indistinguishable from *Nlgn3^+/+^* mice in the time spent in contact with an unfamiliar female [*Nlgn3^+/+^* 99.8 ± 8.36 s, *Nlgn3^+/–^*_WT_ 114.1 ± 6 s, *Nlgn3^+/–^*_KO_ 68 ± 3.8 s, *Nlgn3^–/–^* 65.8 ± 6.3 s, P < 0.0001, *F*_(3,33)_ = 12.5; Fig. 7*A*], distance traveled in the OF [*Nlgn3^+/+^* 4515 ± 321 cm, *Nlgn3^+/–^*_WT_ 4042 ± 206 cm, *Nlgn3^+/–^*_KO_ 4398 ± 508 cm, *Nlgn3^–/–^* 5870 ± 274 cm, P = 0.0011; Fig. 7*B*], thigmotaxis [*Nlgn3^+/+^* 0.23 ± 0.02, *Nlgn3^+/–^*_WT_ 0.25 ± 0.01, *Nlgn3^+/–^*_KO_ 0.24 ± 0.02, *Nlgn3^–/–^* 0.31 ± 0.01, P = 0.0009, *F*_(3,45)_ = 6.5; Fig. 7*C*], and time spent in the open arm of the EPM (*Nlgn3^+/+^* 130.4 ± 7.1 s, *Nlgn3^+/–^*_WT_ 109.3 ± 8.8 s, *Nlgn3^+/–^*_KO_ 127.6 ± 9.1 s, *Nlgn3^–/–^* 124 ± 13.3 s; Fig. 7*D*). However, *Nlgn3^–/–^* mice showed less time spent in contact with an unfamiliar female, decreased thigmotaxis, and similar amount of time in the open arms of the EPM compared with *Nlgn3^+/+^* mice (Fig. 7*A–D*), showing that the behavior of adult *Nlgn3^–/–^* mice phenocopies that of adult *Nlgn3^y/–^* mice. Interestingly, *Nlgn3^+/–^* mice raised with *Nlgn3^–/–^* mice spent less time in contact with an unfamiliar female than *Nlgn3^+/–^* mice raised with *Nlgn3^+/+^* littermates (Fig. 7*A*), indicating that, like males, female *Nlgn3^–/–^* mice modify the behavior of their littermates. Note that in measures of anxiety, the *Nlgn3^+/–^* mice raised with *Nlgn3^–/–^* mice were indistinguishable from *Nlgn3^+/–^* mice raised with *Nlgn3^+/+^* littermates (Fig. 7*B–D*), suggesting that the behavior of *Nlgn3^–/–^* mice does not affect the anxiety of their littermates. These results demonstrate that the behavior of adult *Nlgn3^–/–^* mice phenocopies that of *Nlgn3^y/–^* adult mice and that *Nlgn3^–/–^* mice have a different influence on the social behavior of their littermates than *Nlgn3^+/+^* mice.

## Discussion

Taken together, the results of these experiments reveal an unexpected impact of the *Nlgn3* deletion on social behavior in male and female mouse littermates. First, we showed that young and adult male *Nlgn3^y/+^* and *Nlgn3^y/–^* mice modify each other’s social behavior. In addition, our results indicate that MGH increases adult *Nlgn3^y/–^* mouse anxiety, related to novel environment but not to height, and potentially increases their compulsive behavior. This result is consistent with a previous report showing that the lack of *Nlgn3* affects only specific aspects of anxiety in mice (Radyushkin et al., 2009). Importantly, we found that re-expression of *Nlgn3* in *Pvalb*-expressing interneurons was sufficient to normalize the behavior of *Nlgn3^y/–^* mice and restore normal social behavior in their wild-type littermates, demonstrating that *Nlgn3^y/–^* mice are causing this phenotype in their *Nlgn3^y/+^* littermates. Finally, although the behavior of *Nlgn3^–/–^* mice is not modified by the social environment, we observed that housing with *Nlgn3^+/+^* or *Nlgn3^–/–^* mice promotes different interests in social behavior in *Nlgn3^+/–^* mice.

Using *Nlgn3^y/–^Pvalb^Cre/+^* mice, we were able to demonstrate a causal link between the behavioral phenotype of *Nlgn3^y/–^* mice and that of their littermates. Some evidence confirms that the phenotype of *Nlgn3^y/+^* mice in MGH cannot be due to mothering or early life events. First, all dams of *Nlgn3^y/+^*, *Nlgn3^y/–^*, *Nlgn3^y/+^Pvalb^Cre/+^*, and *Nlgn3^y/–^Pvalb^Cre/+^* mice were of the same *Nlgn3^+/–^* genotype. We found that the maternal behavior of these *Nlgn3^+/–^* dams is comparable to that of *Nlgn3^+/+^* mice (data not shown), consistent with an absence of mothering effect. Second, activation of the *Pvalb* promoter in the brain occurs around P14 (http://developingmouse.brain-map.org); therefore, the interference of behavior between *Nlgn3^y/+^* mice and their *Nlgn3^y/–^* littermates is unlikely to be due to prenatal events. Third, transfer of anxiety or fear (Langford et al., 2006; Smith et al., 2016) could explain the phenotype observed in *Nlgn3^y/+^* mice in MGH. However, we did not find any increased anxiety in young animals or adult female mice, showing that the defective social behavior observed in *Nlgn3^y/+^* mice from MGH is not due to social anxiety as a result of MGH. Finally, re-expression of *Nlgn3* in parvalbumin-expressing cells restores the social behavior of *Nlgn3^y/–^* mice and that of their *Nlgn3^y/+^* littermates without affecting the hyperactivity of *Nlgn3^y/–^* mice. This result suggests that it is not the hyperactivity of *Nlgn3^y/–^* mice that causes the social phenotype in *Nlgn3^y/+^* mice but rather their social behavior deficits.

The finding that *Nlgn3* in parvalbumin cells controls social behavior is consistent with studies showing that mice lacking parvalbumin display deficits in social behavior (Wöhr et al., 2015). Loss of parvalbumin also leads to defects in excitation and inhibition (E/I) balance, a common pathologic feature in mouse models of ASD (Lee et al., 2017). Results show that the phenotype of *Nlgn3^y/–^* mice is also underlined by a deficit in E/I balance caused by a decrease in inhibitory but not excitatory postsynaptic currents in the striatum (Rothwell et al., 2014). Re-expression of *Nlgn3* in parvalbumin-expressing interneurons is therefore likely to be sufficient to restore inhibitory postsynaptic currents and E/I balance in neuronal circuits, rescuing some aspect of the Nlgn3^y/–^ mouse phenotype. Theories on the pathophysiology of ASD have hypothesized that some of the symptoms associated with ASD may be caused by an E/I imbalance in the cortex or the striatum (Rapanelli et al., 2017). In *Nlgn3^y/–^Pvalb^Cre/+^* mice, restoration of the E/I balance is more likely to occur in the striatum, where we detected re-expression of *Nlgn3*, rather than in the cortex, where we detected low levels of re-expression, close to background. We also detected a high level of re-expression of *Nlgn3* in the cerebellum, a brain region associated with the *Nlgn3^y/–^* mouse phenotype (Baudouin, 2014), ASD-associated phenotypes in mice (Tsai et al., 2012), and symptoms of ASD in humans (Wang et al., 2014). The alleviation of the phenotype in *Nlgn3^y/–^* mice could therefore arise from a restoration of neuronal network activity in the striatum or cerebellum.

Our results also show similarities in phenotypes of male and female mice lacking *Nlgn3*. Indeed, like the *Nlgn3^y/–^* mice studied here and in Radyushkin et al. (2009), *Nlgn3^–/–^* mice show increased activity in the OF and spend similar amounts of time in the open arms of the EPM compared with wild-type mice. Unlike *Nlgn3^–/–^* mice, *Nlgn3^+/–^* mice show behavior similar to that of their *Nlgn3^+/+^* littermates. These results demonstrate that the effect of a loss-of-function mutation in *Nlgn3* is dose dependent and, because *Nlgn3* is an X-linked gene, less likely to affect females than males. In addition, *Nlgn3^–/–^* mice are likely modifying the social behavior of their littermates, suggesting that, as in males, social hierarchy or at least group inequality may exist in female laboratory mice. In contrast to *Nlgn3^y/–^* mice, young *Nlgn3^–/–^* mice show no sensitivity to the social environment. Importantly, these results indicate that the social environment has a sexually dimorphic effect on the phenotypes associated with ASD, which could in part explain the increased prevalence in boys versus girls in humans.

Our observation that the behavior of transgenic mice can affect that of their wild-type littermates, and vice versa, is a crucial parameter to take into account in the interpretation of experiments. Much evidence indicates that such a phenomenon may not be limited to *Nlgn3^y/–^* mice but could extend to other models. First, several genetic mouse models of ASD display social submission (Spencer et al., 2005; Yang et al., 2015a, 2015b) or courtship behavior deficits, potentially indicative of a social dominance phenotype (Kazdoba et al., 2016). Raising these mice with wild-type littermates could create similar social heterogeneity, and the associated phenotypes, to that of *Nlgn3^y/–^* mice and their wild-type littermates. Second, the behavioral phenotype of *Nlgn3^y/–^* is associated with dysfunctions of *Pvalb*-expressing interneurons [Polepalli et al. (2017) and Figs. 4 and 5] and type 1 mGluRs (Baudouin et al., 2012). Several studies have suggested that, despite the heterogeneity of genetic factors causing ASD, similar pathophysiological phenotypes converge on common behavioral phenotypes (Gogolla et al., 2009, 2014; Auerbach et al., 2011; Baudouin, 2014). Several mouse models of ASD show cellular (Gogolla et al., 2014; Karayannis et al., 2014; Ito-Ishida et al., 2015) and molecular (Bear et al., 2004; Tian et al., 2015; Wang et al., 2016) pathophysiology similar to *Nlgn3^y/–^* mice, associated with a common social behavior phenotype; therefore, these transgenic mice could also modify the behavior of their wild-type littermates. Our observation that the phenotype of *Nlgn3^y/–^* mice modifies the social behavior of their *Nlgn3^y/+^* littermates implies that using wild-type littermates as unique controls for experiments with animal models may lead to false interpretations of results. Therefore, these findings could profoundly affect the interpretation of experiments using mouse models of ASD, and we strongly recommend that standard laboratory practice should take into account the potential confounding effect of social heterogeneity by including independent nonlittermate wild-type mice as additional controls.

## Synthesis

Reviewing Editor: Carmen Sandi, Swiss Federal Institute of Technology Decisions are customarily a result of the Reviewing Editor and the peer reviewers coming together and discussing their recommendations until a consensus is reached. When revisions are invited, a fact-based synthesis statement explaining their decision and outlining what is needed to prepare a revision will be listed below. The following reviewer(s) agreed to reveal their identity: Stamatina Tzanoulinou, Ewelina Knapska.

The idea that co-housing of transgenic and wild-type mice changes behavior of mice from a particular genotype has not as yet been well explored. The subject is important because such influence can have important methodological significance regarding social housing conditions of experimental animals.

The authors have made an important effort to address the issues raised after the first submission. They have now included an illustration of the breeding schemes and added details to the figure legends that is helpful to grasp the information readily. They have modified the introduction and discussion according to the reviewers and editor requests, and thus, the manuscript is more coherent and complete. However, there are still the following minor issues that need to be addressed:

1. In Figures 5 and 6, anxiety-like behaviors of the different mouse genotypes are investigated. Authors should emphasize in the text that the anxiety behavior measured in figure 6 with the marbles test is assessing a different kind of anxiety-like behavior. Marble burying is also considered by some researchers to reflect compulsive-like behavior rather than anxiety. Thus, authors should discuss this possibility, especially because there were no differences in the EPM.

2. In Figures 4D, 6E, statistical differences between single groups should be shown - it will clarify the effects of rescue for both genotypes.

3. Lines 76-77 - a part of the sentence seems to be missing.

4. Line 193 - `systematically' seems to be an editing error.

5. Line 249 - 17.8 s is not consistent with the data showed in the respective fig.

6. Line 390 -`between' seems to be an editing error.

7. Line 465 - 0.04 is given twice.

8. Line 470 - the same genotype is given twice in the comparison.

9. The discussion (line 363) indicates that social submission in Nlgn3y/- is associated with an increase in their anxiety and reaction to a novel environment. However, there are no correlational analyses to prove this point. This sentence should be changed to better representing the data included.
